# Noninvasive Oral Hyperspectral Imaging–Driven Digital Diagnosis of Heart Failure With Preserved Ejection Fraction: Model Development and Validation Study

**DOI:** 10.2196/67256

**Published:** 2025-01-07

**Authors:** Xiaomeng Yang, Zeyan Li, Lei Lei, Xiaoyu Shi, Dingming Zhang, Fei Zhou, Wenjing Li, Tianyou Xu, Xinyu Liu, Songyun Wang, Quan Yuan, Jian Yang, Xinyu Wang, Yanfei Zhong, Lilei Yu

**Affiliations:** 1 Cardiovascular Hospital Renmin Hospital of Wuhan University Wuhan China; 2 Hubei Key Laboratory of Autonomic Nervous System Modulation Wuhan University Wuhan China; 3 Cardiac Autonomic Nervous System Research Center Wuhan University Wuhan China; 4 State Key Laboratory of Information Engineering in Surveying, Mapping and Remote Sensing Wuhan University Wuhan China; 5 College of Geomatics Xi'an University of Science and Technology Xi'an China; 6 Department of Cardiology The First College of Clinical Medical Science Yichang Central People's Hospital Yichang China; 7 Hubei Key Laboratory of Ischemic Cardiovascular Disease China Three Gorges University Yichang China; 8 Medical Remote Sensing Information Cross-Institute Wuhan University Wuhan China; 9 College of Chemistry and Molecular Sciences Key Laboratory of Biomedical Polymers of Ministry of Education Wuhan University Wuhan China; 10 lnstitute of Molecular Medicine Renmin Hospital of Wuhan University Wuhan China; 11 School of Remote Sensing and Information Engineering Wuhan University Wuhan China

**Keywords:** heart failure with preserved ejection fraction, HFpEF, hyperspectral imaging, HSI, diagnostic model, digital health, Shapley Additive Explanations, SHAP, machine learning, artificial intelligence, AI, cardiovascular disease, predictive modeling, oral health

## Abstract

**Background:**

Oral microenvironmental disorders are associated with an increased risk of heart failure with preserved ejection fraction (HFpEF). Hyperspectral imaging (HSI) technology enables the detection of substances that are visually indistinguishable to the human eye, providing a noninvasive approach with extensive applications in medical diagnostics.

**Objective:**

The objective of this study is to develop and validate a digital, noninvasive oral diagnostic model for patients with HFpEF using HSI combined with various machine learning algorithms.

**Methods:**

Between April 2023 and August 2023, a total of 140 patients were recruited from Renmin Hospital of Wuhan University to serve as the training and internal testing groups for this study. Subsequently, from August 2024 to September 2024, an additional 35 patients were enrolled from Three Gorges University and Yichang Central People’s Hospital to constitute the external testing group. After preprocessing to ensure image quality, spectral and textural features were extracted from the images. We extracted 25 spectral bands from each patient image and obtained 8 corresponding texture features to evaluate the performance of 28 machine learning algorithms for their ability to distinguish control participants from participants with HFpEF. The model demonstrating the optimal performance in both internal and external testing groups was selected to construct the HFpEF diagnostic model. Hyperspectral bands significant for identifying participants with HFpEF were identified for further interpretative analysis. The Shapley Additive Explanations (SHAP) model was used to provide analytical insights into feature importance.

**Results:**

Participants were divided into a training group (n=105), internal testing group (n=35), and external testing group (n=35), with consistent baseline characteristics across groups. Among the 28 algorithms tested, the random forest algorithm demonstrated superior performance with an area under the receiver operating characteristic curve (AUC) of 0.884 and an accuracy of 82.9% in the internal testing group, as well as an AUC of 0.812 and an accuracy of 85.7% in the external testing group. For model interpretation, we used the top 25 features identified by the random forest algorithm. The SHAP analysis revealed discernible distinctions between control participants and participants with HFpEF, thereby validating the diagnostic model’s capacity to accurately identify participants with HFpEF.

**Conclusions:**

This noninvasive and efficient model facilitates the identification of individuals with HFpEF, thereby promoting early detection, diagnosis, and treatment. Our research presents a clinically advanced diagnostic framework for HFpEF, validated using independent data sets and demonstrating significant potential to enhance patient care.

**Trial Registration:**

China Clinical Trial Registry ChiCTR2300078855; https://www.chictr.org.cn/showproj.html?proj=207133

## Introduction

About one-half of patients with chronic heart failure have heart failure with preserved ejection fraction (HFpEF), which has received wide attention in recent years and poses a serious threat to the management of patient health [1,2]. The pathogenesis of HFpEF is complex, and the pathological mechanism is still unclear, so a swift, noninvasive diagnostic strategy is still lacking [3]. Early diagnosis of HFpEF is often difficult because the left ventricular ejection fraction of patients with HFpEF is within the normal range and the symptoms of heart failure are often nonspecific. The diagnosis of HFpEF depends on clinical symptoms, laboratory tests, echocardiography, and an invasive hemodynamics test [4]. However, descriptions of clinical symptoms are subjective, and different clinical symptoms cannot be measured using a unified standard. Echocardiography and hemodynamics tests need to be performed by experienced clinicians in hospitals, thus limiting early identification and timely diagnosis of HFpEF, which introduces obstacles to the health management of individuals with HFpEF [5].

The health of the oral environment has an important impact on cardiovascular health, and oral microenvironmental disorders have been associated with an increased risk of HFpEF [6]. Poor oral health may cause an inflammatory response that is strongly associated with heart failure [7,8]. The tongue, as an important part of the oral cavity, plays an important role in oral health, and the dorsum of the tongue carries the largest number of microbial species, which is an important part of oral health [9]. Inflammation and oxidative stress in the body caused by alterations in the oral microbiome are associated with the risk of developing heart failure [10]. Observing the oral microenvironment of individuals with HFpEF can reflect their lifestyle, dietary habits, and intestinal health [11]. An automated device or system that allows doctors to perform a quick tongue-based diagnosis would be helpful in clinical practice.

Light, which is a single color before dispersion, becomes a pattern of colors arranged in order of wavelength after dispersion; this is called the optical spectrum or optical band [12]. Hyperspectral imaging (HSI) consists of narrower bands, is a technique that captures and analyzes the details of each band in a region, can therefore detect substances that are visually indistinguishable to humans, and has a wide range of applications in many fields [13]. In recent years, many studies have explored the application of HSI technology in medicine [14]. The emergence of handheld HSI cameras, which allow users to quickly capture spectral images, has boosted the application of spectral imaging in medicine [15]. HSI technology has been used in patient information acquisition, medical image analysis, and disease diagnosis [16,17]. HSI is expected to promote the management of patient health. Notably, numerous innovative heart failure prediction models have been developed using common clinical indicators and advanced machine learning techniques [18-23]. These studies primarily focused on the prognosis of heart failure or enhanced existing diagnostic modalities such as echocardiography [24-26]. There exists a significant need for the development of noninvasive, easily accessible diagnostic tools specifically targeting HFpEF, which is a subtype of heart failure characterized by subtle clinical manifestations and complex pathophysiology.

In this study, we collected HSI information of the oral environment, and multiple algorithms were used to select the most characteristic spectral bands of individuals with HFpEF. The best model was selected for internal and external testing, and we used the Shapley Additive Explanations (SHAP) model to additively interpret the best model. The digital HSI HFpEF diagnostic model constructed in this study can help with early detection and management of individuals with HFpEF.

## Methods

### Study Populations

**Figure 1:**
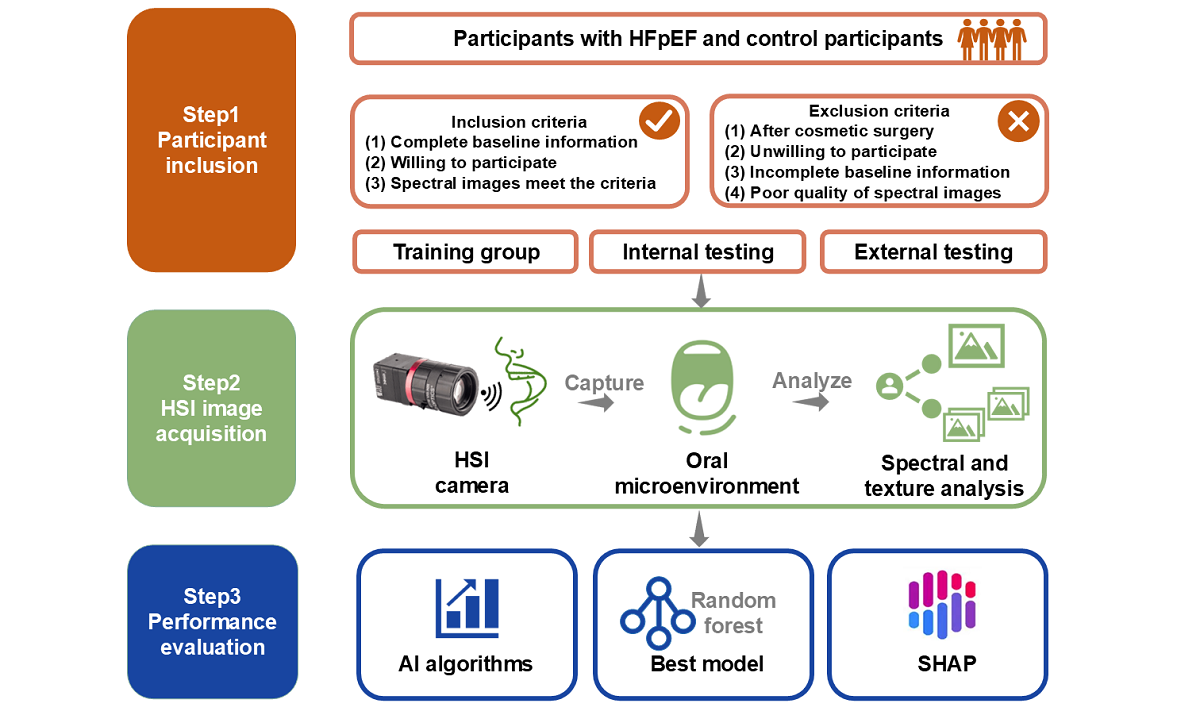
Study protocol. AI: artificial intelligence; HFpEF: heart failure with preserved ejection fraction; HSI: hyperspectral imaging; SHAP: Shapley Additive Explanations.

We included 196 patients who visited Renmin Hospital Wuhan University from April 2023 to August 2023; they comprised the training group and internal testing groups. We included 53 patients who visited Yichang Central People’s Hospital from August 2024 to September 2024 as the external testing group (Figure 1). For all participants, HSI and clinical information were collected, and routine tests were conducted. Participants were selected according to the inclusion and exclusion criteria. HFpEF was diagnosed using international standards [4], including (1) typical signs and symptoms of heart failure; (2) left ventricular ejection fraction ≥50%, as assessed by echocardiography performed by a proficient physician; and (3) brain natriuretic peptide >35 ng/L or amino-terminal pro-brain natriuretic peptide >125 ng/L. In addition to these criteria, participants also had to have at least left ventricular hypertrophy or left atrial enlargement with abnormal diastolic function. Individuals were excluded for any of the following reasons: previous cosmetic surgery and facial aesthetics, severe hepatic or renal organ insufficiency, mental or legal incapacity, malignancy-related diseases, other diseases such as psychiatric or neurological disorders, and unable to complete the study activities. Participants were consecutively included in the training and internal testing groups (140 of 196 participants) before randomization using a ratio of 3:1, while participants in the external testing group (35 of 53 participants) were also consecutively included [19]. The manuscript was written in strict accordance with the Guidelines for Developing and Reporting Machine Learning Predictive Models in Biomedical Research [20,21].

### Ethical Considerations

All participants were informed about the study and signed an informed consent document. The study protocol was reviewed and approved by the Ethics Committee of Renmin Hospital of Wuhan University (number WDRM2023-K174) and Yichang Central People’s Hospital (number 2024-216-01).

### HSI Collection

**Figure 2:**
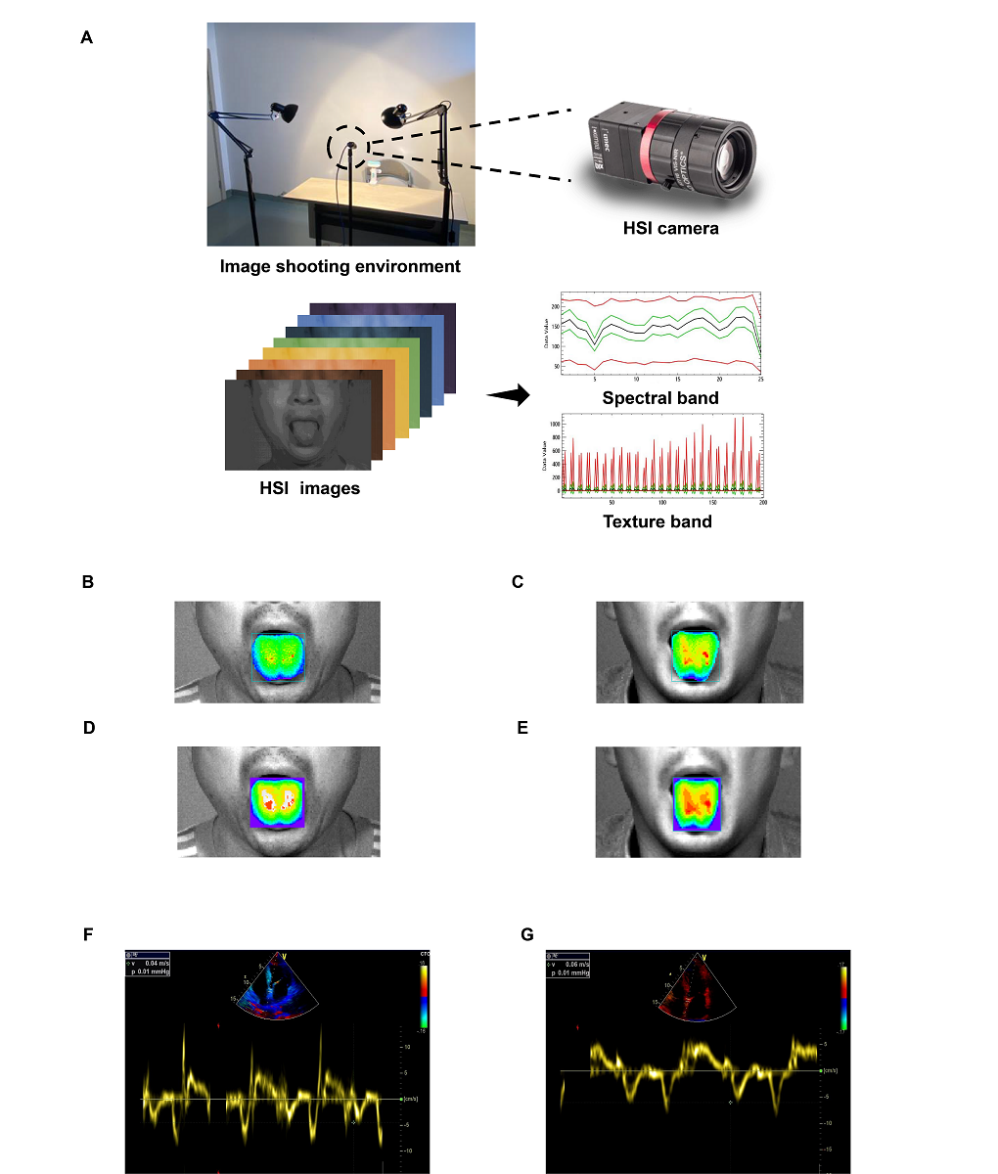
(A) Hyperspectral imaging (HSI) acquisition environment, (B) processing of a spectral image for a control participant, (C) processing of a spectral image for a participant with heart failure with preserved ejection fraction (HFpEF), (D) processing of a texture image for a control participant, (E) processing of a texture image for a participant with HFpEF, (F) representative HSI and echocardiogram of a control participant, and (G) representative HSI and echocardiogram of a participant with HFpEF.

We prepared a room with good light avoidance conditions and created a dark environment using curtains, blackboards, and other items to avoid external light interference as much as possible to ensure that all participants were in the same light environment for image acquisition. A halogen lamp was chosen as the only light source. The participant’s head and face were fixed to ensure that there were no accessories nor hair to obscure facial features. The distance between the HSI camera and the participants was maintained to ensure that the size of the facial features was in the same area of each spectral image and that all participants had the same image size and clarity. We asked participants to expose their tongues for HSI acquisition (Figure 2A). Each participant was provided with a single hyperspectral photomicrograph, and only 1 oral spectral image meeting the criteria was captured.

An HSI camera (MQ0220HG-IM-SM4X4-VIS; XIMEA) captured 25 spectral bands in the 665 nm to 960 nm spectral range. The camera has a pixel resolution of 644 × 484 (black and white) or 640 × 480 (color), pixel size of 7.4 × 7.4 µm, active area size of 4.8 × 3.6 mm, sensor diagonal of 5.9 mm, and dynamic range of 60 dB. The camera has an exposure time of 54 µs to 1 s with a step of 7.56 µs, with a capture time less than 1 s per image (Figure 2A). We checked image quality after image capture to ensure that all images had the same luminance, were in sharp focus, and were of good quality.

### HSI Analysis

We preprocessed the obtained images [22,23]. HSI preprocessing methods are of great importance in HSI analysis. Effective preprocessing methods can minimize or even eliminate the influence of extraneous information (eg, sample background, electrical noise, stray light). We first checked the image quality of all oral images, and when poor image quality was found, the participant’s oral images were taken repeatedly in the same environment to ensure that the images could be qualified and used for further analysis. We preprocessed the images using the median filtering method to reduce any noise. HSI was preprocessed using a normalized method to reduce redundant information from the original bands and improve the precision of the HSI [27].

All images were edited using image analysis software (ENVI [Environment for Visualizing Images], version 5.3; NV5 Geospatial Solutions Inc). Backgrounds and clothing were cropped out, and the tongue images were retained. The spectral data of the features were initially extracted from the cropped images to obtain the mean and SD for each spectral photograph. Each spectral image was capable of extracting information for 25 spectral bands. Subsequently, texture analysis was conducted on the image, resulting in the generation of 8 texture features based on the mean and SD values for each band. For each participant, their spectral image could be obtained with 50 original band values and 400 band texture values.

### Machine Learning Methods

In order to find the most suitable algorithm for the model, we used as many of the currently known machine learning algorithms as possible, resulting in a total of 28 machine learning algorithms. The single algorithms included linear models, such as linear regression, logistic regression, least absolute shrinkage and selection operator (LASSO) regression, ridge regression, and ElasticNet regression. Other types of single algorithms were also used such as decision tree algorithms, neural network algorithms, and support vector machine (SVM) algorithms. The ensemble models included both a boosting algorithm and bagging algorithm; the boosting algorithms included XGBoost algorithms, and the bagging models included random forest algorithms. For probabilistic models, we used Bayesian algorithms, Bernoulli naïve Bayes algorithms, and Gaussian naïve Bayes algorithms.

We performed 5-fold cross-validation in the training group, which was randomly divided into 5 subsets, each with the same sample size. The model was constructed using 4 subsets, and the model performance was assessed using the remaining data. Next, the model was constructed with another combination of the 4 subsets, and the model performance was assessed using the remaining data. The 5 cycles were performed in sequence, and the results obtained for the model performances were summarized. After constructing the model in the training group, model performance was evaluated in the testing group. We evaluated the importance of features using a random forest variable importance ranking method, where the importance of a feature is typically measured by calculating how often the feature is used in a decision tree: the more often a feature is used in the decision tree or the more it contributes to dividing the data, the higher its importance score. The results of all the decision trees’ assessments of feature importance were summarized to obtain an importance score for each feature in the entire random forest model. Each machine learning model was constructed by incorporating these rankings until the performance of the model could not be improved; the incorporation of features was then stopped, enabling the construction of the model [28].

### Evaluation of Model Performance

Based on the previously mentioned diagnostic criteria for HFpEF, we categorized all participants using a bivariate category. During the model construction process, we evaluated the ability of each model to be able to distinguish between participants with HFpEF and control participants. We evaluated the performance of each model by comparing the ability of the model to accurately identify participants with HFpEF. We assessed the performance of all algorithms; the accuracy, sensitivity, specificity, *F*_1_-score, positive predictive value (PPV), and negative predictive value (NPV) of each algorithm were calculated separately. The results are presented in a table. Receiver operating characteristic (ROC) curves of the 5 best performing models for the internal testing and external testing groups were drawn, and the area under the ROC curve (AUC) was calculated. Decision curve analysis (DCA) of the 5 best performing models was implemented to assess the clinical usefulness of the models. Calibration curves of the 5 best performing models were plotted to assess the calibration.

### SHAP Model Interpretation

We used the SHAP model to explain the best machine learning algorithm [29]. SHAP is a method of interpreting the output of a machine learning model and assigns weights to the optimal indexes using the Shapley values derived from the analysis; we used it to quantify the contribution of different features to the predicted values [30]. The SHAP value allows visual identification of the impact of different features on the model prediction results. In addition, the SHAP value explains the prediction results for each individual in the training group, helping to understand why the model made a particular prediction. It is also possible to perform an aggregated global interpretation of Shapley values for multiple data points in order to provide a total interpretation of the model and to demonstrate the interconnections between different features. SHAP analysis was implemented using Python software, and the results are presented using visualization methods. We first demonstrated the contribution of the bands selected by the best model and ranking by the contribution of different features. We then ranked the contribution of each individual in the study to the model and showed the SHAP analysis results for 1 participant with HFpEF and 1 control participant using intuitive visualization methods to reveal the contribution of different features.

### Statistical Analysis

The data in this study were analyzed using SPSS 23.0 (IBM Corp). Model construction and graphic drawing were completed using R version 3.6.1 (R Foundation for Statistical Computing). We used the Shapiro-Wilk normality test to check the distribution of the data. A 1-way ANOVA was used to compare continuous variables that had a normal distribution, and the results are shown as mean (SD). The Kruskal-Wallis *H* test was used to compare continuous variables with nonnormal distributions, and the results are shown as median (IQR). The Fisher exact test was used to compare categorical data, and the results are reported as counts and percentages. *P*<.05 was considered statistically significant.

## Results

### Study Population

Individuals who visited Renmin Hospital of Wuhan University from April 2023 to July 2023 were enrolled. A total of 196 participants were included in this study after excluding individuals who did not meet the inclusion criteria, had incomplete baseline data, did not cooperate with spectral acquisition, or had poor image quality. Data were collected for a total of 140 participants in the training and testing groups (Figure 1). Individuals who visited Yichang Central People’s Hospital from August 2024 to September 2024 were also enrolled. A total of 53 participants were included in this study after excluding individuals who did not meet the inclusion criteria, had incomplete baseline data, did not cooperate with spectral acquisition, or had poor image quality. Data were collected for a total of 35 patients in the external testing groups (Figure 1). The participants were divided into training (n=105), internal testing (n=35), and external testing (n=35) groups. The baseline information, which included basic information, previous medical history, and basic examination and test results, for the training and testing groups was compared (Table 1). There were no significant differences in the baseline data between the training, internal testing, and external testing groups. 

**Table 1:** Participants’ baseline characteristics.

Characteristic			Training group (n=105)		Internal testing group (n=35)		External testing group (n=35)		*P* value	
Age (years), mean (SD)			62 (11)		64 (11)		59 (15)		.20	
**Sex, n (%)**										.11
	Male	64 (61)		28 (80)		24 (68.6)				
	Female	41 (39.1)		7 (20)		11 (31.4)				
**Current smoker, n (%)**										.32
	No	39 (37.1)		18 (51.4)		15 (42.9)				
	Yes	66 (62.9)		17 (48.6)		20 (57.1)				
**Current drinker, n (%)**										.94
	No	24 (22.9)		8 (22.9)		9 (25.7)				
	Yes	81 (77.1)		27 (77.1)		26 (74.3)				
**Hypertension**										.35
	No	53 (50.5)		20 (57.1)		14 (40)				
	Yes	52 (49.5)		15 (42.9)		21 (60)				
**Diabetes**										.61
	No	26 (24.8)		9 (25.7)		6 (17.1)				
	Yes	79 (75.2)		26 (74.3)		29 (82.9)				
CK-MB^a^ (ng/mL), median (IQR)			1.34 (0.59-2.49)		1.62 (0.75-2.34)		1.75 (1.33-2.49)		.22	
Cardiac troponin I (ng/mL), median (IQR)			1.45 (0.06-2.90)		1.92 (0.78-4.08)		2.05 (0.31-2.76)		.18	
TG^b^ (mmol/L), median (IQR)			1.43 (1.10-1.87)		1.57 (1.23-2.01)		1.68 (1.29-2.20)		.14	
TC^c^ (mmol/L), mean (SD)			3.68 (1.17)		3.59 (1.17)		3.57 (1.04)		.85	
HDL-C^d^ (mmol/L), median (IQR)			1.30 (1.06-2.00)		1.20 (0.92-1.66)		1.67 (1.25-2.10)		.08	
LDL-C^e^ (mmol/L), median (IQR)			2.21 (1.46-2.70)		2.11 (1.53-2.34)		2.58 (1.80-3.27)		.052	
TSH^f^ (μIU/mL), median (IQR)			1.97 (1.32-3.02)		1.78 (1.09-2.71)		1.77 (1.20-2.46)		.34	
FT3^g^ (pg/mL), median (IQR)			3.25 (2.96-3.58)		3.17 (2.72-3.58)		3.30 (3.09-3.54)		.62	
FT4^h^ (ng/dL), median (IQR)			1.19 (1.04-1.34)		1.23 (1.07-1.37)		1.19 (1.08-1.29)		.48	
BMI (kg/m^2^), median (IQR)			24.6 (22.6-26.7)		25.4 (23.7-27.5)		24.7 (22.1-26.8)		.47	
NT-proBNP^i^ (pg/mL), median (IQR)			95 (60-375)		153 (74-271)		86 (52-114)		.23	
**HFpEF^j^, n (%)**										.69
	No	74 (70.5)		24 (68.6)		27 (77.1)				
	Yes	31 (29.5)		11 (31.4)		8 (22.9)				
LAD^k^ (mm), median (IQR)			37.0 (34.0-41.0)		36.0 (31.5-39.0)		36.0 (33.0-39.0)		.38	
RAD^l^ (mm), median (IQR)			35.0 (33.0-38.0)		34.0 (31.5-36.5)		35.0 (33.5-38.0)		.24	
LVEF^m^ (%), median (IQR)			55.0 (51.3-60.0)		58.0 (52.0-60.0)		56.0 (51.0-58.0)		.07	
LVDD^n^ (mm), median (IQR)			49.0 (46.0-52.0)		48.0 (45.5-50.0)		49.0 (45.0-51.5)		.25	
E/e′ ratio, median (IQR)			10.7 (8.3-15.3)		11.6 (9.5-16.3)		10.2 (7.9-14.2)		.26	

^a^CK-MB: creatine kinase-MB.

^b^TG: triglyceride.

^c^TC: total cholesterol.

^d^HDL-c: high-density lipoprotein cholesterol.

^e^LDL-c: low density lipoprotein cholesterol.

^f^TSH: thyroid-stimulating hormone.

^g^FT3: free triiodothyronine.

^h^FT4: free thyroxine.

^i^NT-proBNP: amino-terminal pro-brain natriuretic peptide.

^j^HFpEF: heart failure with preserved ejection fraction.

^k^LAD: left atrium diameter.

^l^RAD: right atrium diameter.

^m^LVEF: left ventricular ejection fraction.

^n^LVDD: left ventricular end diastolic diameter.

### Image Processing and Machine Learning Algorithm Comparison

**Figure 3:**
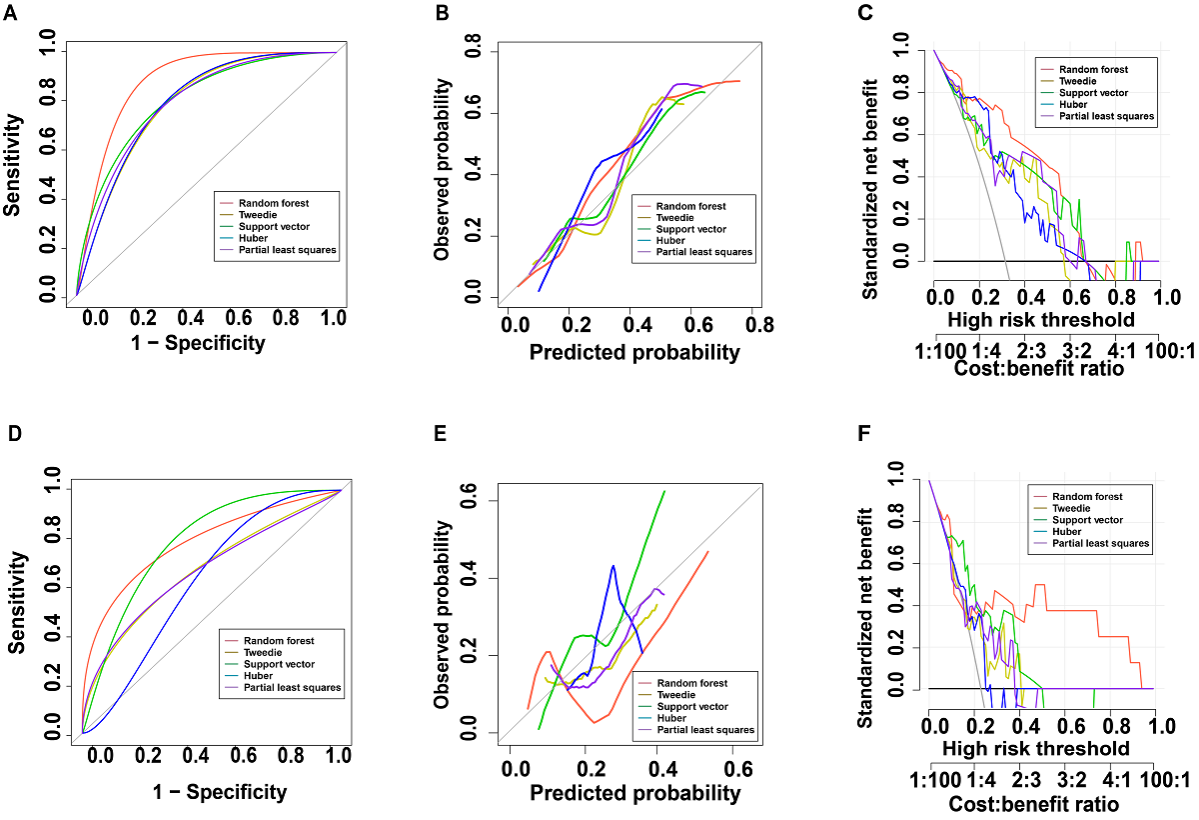
(A) Receiver operating characteristic (ROC) curve, (B) calibration curve, and (C) decision curve analysis (DCA) for the internal testing group and (D) ROC curve, (E) calibration curve, and (F) DCA for the external testing group.

HSI data were collected for the training, internal testing, and external testing groups. Poor image quality for 4 participants in the training group, 1 participant in the internal testing group, and 2 participants in the external testing group made analysis difficult. Therefore, we acquired oral HSI in the same environmental conditions for those participants again and used those images for the next analysis after ensuring that the image quality met the standards. After preprocessing the images, spectral values and texture values of the images were extracted. We present the characteristic tongue HSI and echocardiography images of control participants and participants with HFpEF in Figures 2B-2G. We used 28 algorithms to filter the characteristic spectral bands of images from participants with HFpEF. We used 5-fold cross-validation in the training group, and the model performance was evaluated in the testing group. The accuracy, *F*_1_-score, PPV, NPV, sensitivity, specificity, and AUC of the different algorithms were calculated separately, and the results for the 28 algorithms are listed in Table S1 in Multimedia Appendix 1. Among all the models constructed, the Tweedie, SVM, partial least squares, Huber, and random forest algorithms were the top 5 in terms of performance, showing good ability to identify participants with HFpEF. The ROC curves (Figure 3A), calibration curves (Figure 3B), and DCA (Figure 3C) of the top 5 algorithms were plotted, and the AUCs were calculated (Table S1 in Multimedia Appendix 1). The AUCs in the internal testing group were 0.884 (95% CI 0.769-1.000) for random forest, 0.795 (95% CI 0.633-0.958) for Tweedie, 0.814 (95% CI 0.657-0.972) for SVM, 0.803 (95% CI 0.659-0.947) for Huber, and 0.799 (95% CI 0.640-0.959) for partial least squares.

### External Validation of the Top 5 Algorithms

The HSI collected in the external testing group were preprocessed in the same way, and the spectral values and texture values were extracted and analyzed. We used the top 5 best performance models in the internal testing group to analyze the external testing data. The accuracy, *F*_1_-score, PPV, NPV, sensitivity, specificity, and AUC of the different algorithms were also calculated separately, and the results for the 5 algorithms are listed in Table S2 in Multimedia Appendix 1. The random forest algorithm still had the best model performance among the 5 algorithms. The ROC curves (Figure 3D), calibration curves (Figure 3E), and DCA (Figure 3F) of the 5 algorithms were plotted, and the AUCs were calculated (Table S2 in Multimedia Appendix 1). The AUCs in the external testing group were 0.812 (95% CI 0.633-0.992) for random forest, 0.676 (95% CI 0.438-0.914) for Tweedie, 0.792 (95% CI 0.632-0.951) for SVM, 0.634 (95% CI 0.428-0.841) for Huber, and 0.671 (95% CI 0.428-0.915) for partial least squares. We extracted the features selected by random forest for further interpretation.

### SHAP Interpretation of the Best Algorithms

**Figure 4:**
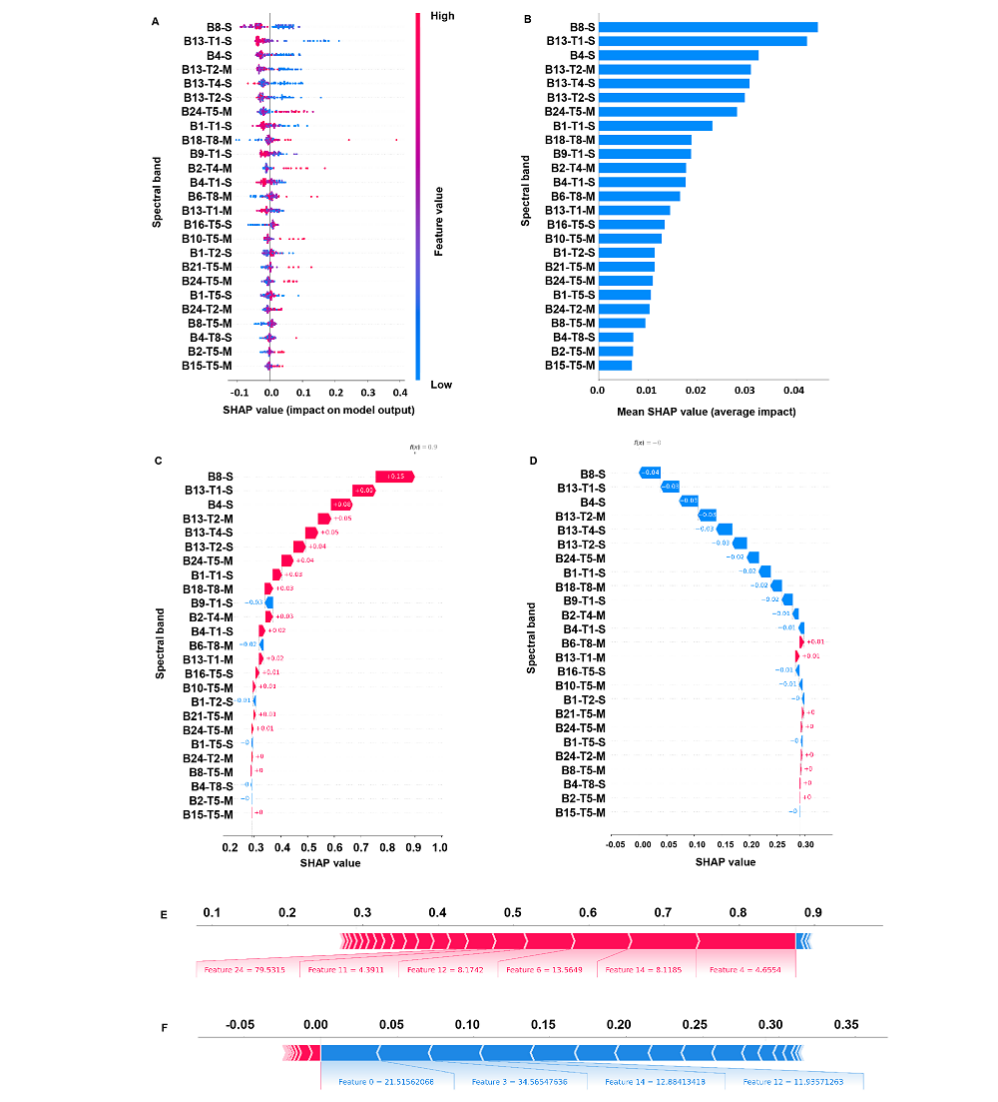
Shapley Additive Explanations (SHAP) of the best algorithm: (A) summary plot, (B) feature importance map, (C) waterfall plot for a control participant, (D) waterfall plot for a participant with heart failure with preserved ejection fraction (HFpEF), (E) bar chart for a control participant, (F) bar chart for a participant with HFpEF.

We used the SHAP model for additive interpretation. The Shapley value was calculated to assign the benefit each characteristic brings to the overall model, showing the contribution of each characteristic to the model’s predicted results. We present the characteristics of the top 5 best performing models in order of their contribution to the overall models in Table S3 in Multimedia Appendix 1. The summary plot (Figure 4A) shows the feature importance ranking and distribution via the Shapley value of each spectral band, where the blue bar indicates that the eigenvalue positively affected the model and the red bar indicates that the eigenvalue negatively affected the model. The Shapley value represents the magnitude of each feature’s impact on the predicted results, with the point farthest from the centerline indicating a greater influence on the model output. The SHAP feature importance map ranks each characteristic by their contribution (Figure 4B), with features at the top having a greater impact on the model output and those near the bottom having a lesser total impact. SHAP waterfall plots and SHAP bar charts (Figure 4C-4F) were used to visualize the Shapley values of individual samples and their individual results. We visualized the model’s ability to recognize HFpEF using a waterfall plot (Figure 4C) and bar chart (Figure 4E) for a control participant and a waterfall plot (Figure 4D) and bar chart (Figure 4F) for a participant with HFpEF. In the waterfall plot, the contribution of each feature is represented by a bar. The length of the bar indicates the magnitude of the feature’s influence on the predicted value. A blue bar indicates that the feature increased the predicted value, and a red bar indicates that the feature decreased the predicted value. There was an intuitive difference between the SHAP results for control participants and those for participants with HFpEF. The HFpEF diagnostic model constructed in this study was able to distinguish control participants from participants with HFpEF.

## Discussion

### Principal Findings

In this study, we innovatively used HSI to acquire oral images of control participants and participants with HFpEF, extracted the spectral and textural information of the HSI, and characterized the HSI using a variety of machine learning algorithms. The optimal algorithm was selected for the construction of the HFpEF diagnostic model, the model performance was validated in the internal and external testing groups, and the SHAP model was used for additive interpretation.

Digital health care is the future of medicine, introducing considerable convenience to the management of people’s health [31]. As artificial intelligence and machine learning continue to advance, enabling automated interpretation and classification of HSI will allow researchers to focus more on deep analysis and decision-making [32]. A large amount of data redundancy is present for HSI data, and traditional image data processing methods struggle to meet processing and analyzing needs; therefore, we generated a series of feature extraction and classification methods for spectral information. The use of multiple artificial intelligence algorithms helps to identify the characteristic bands and textures in large amounts of HSI information [33]. The use of artificial intelligence and machine learning can increase the interpretability of HSI data [34]. To select the most suitable machine learning algorithms for oral spectral images of individuals with HFpEF, we incorporated as many of the current mainstream machine learning models as possible [22]. We analyzed the obtained HSI using 28 machine learning algorithms and filtered the top 5 algorithms for external testing. The random forest algorithm showed good prediction performance in both the internal and external testing groups, so we used the spectral features screened by the random forest algorithm for further analysis. We also performed subgroup analysis based on age and gender in both internal and external testing groups, and the random forest algorithm showed good performance (Figure S1 in Multimedia Appendix 1). Although studies have investigated the use of artificial intelligence for HFpEF diagnosis [19,35,36], these studies primarily focused on constructing echocardiography-based diagnostic models for HFpEF. However, the reliance on specialty physicians to interpret echocardiography exams hinders the early identification of individuals with HFpEF [37]. Moreover, considering the existing diagnostic challenges and limited availability of imaging data during the initial stages of HFpEF, attention should be directed toward noninvasive imaging techniques for diagnosis and management. Therefore, it is imperative to integrate machine learning approaches to develop predictive models for early screening. In our study, we identified spectral bands and textural features that exhibit a close association with the pathophysiological changes observed in HFpEF. This condition is characterized by microvascular dysfunction, inflammation, and oxidative stress leading to alterations in optical properties of oral tissues [3]. HSI effectively captures these subtle changes by detecting variations in light absorption and scattering at different wavelengths. Our model significantly contributes to identifying HFpEF at an early stage, enabling clinicians to promptly initiate appropriate therapies while potentially slowing down disease progression and improving patient health care.

**Figure 5:**
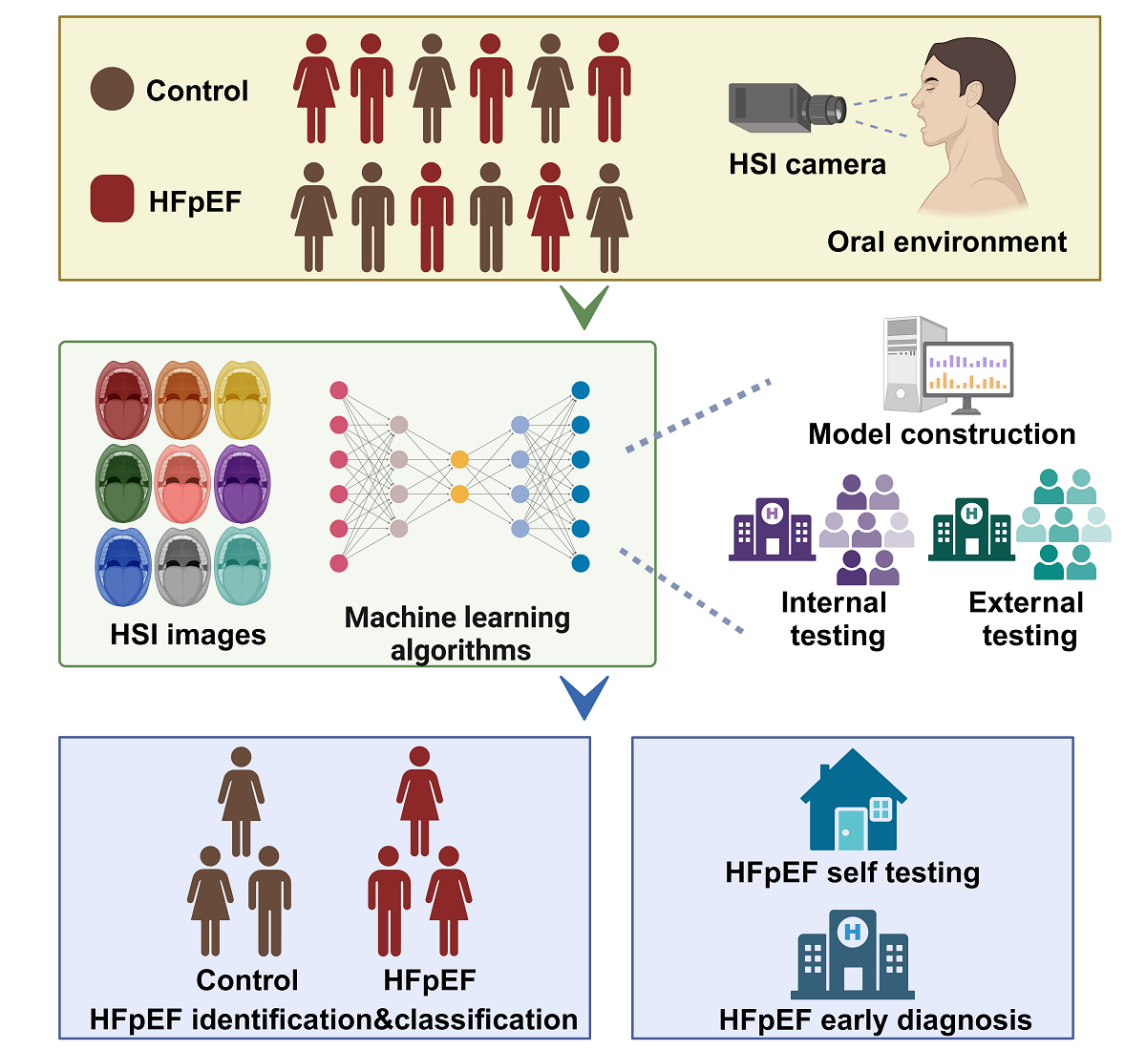
Future application scenarios. HFpEF: heart failure with preserved ejection fraction; HSI: hyperspectral imaging.

To effectively integrate our diagnostic model into existing health care management for HFpEF, we used an additive interpretation of this digital diagnostic model using the SHAP model. This allowed us to evaluate the contribution of each feature to the prediction, quantify the capability of these features to contribute to the overall model, and demonstrate their impact on the final predictive and diagnostic performance [28]. The SHAP model allows each parameter to be analyzed individually, which can be useful in understanding the decisions made by the model and to improve and rationalize the results of the model [38]. In the macrointerpretation of the SHAP model, the ranking of the contribution of the 25 features screened by random forest to the overall model and the ability of each feature to explain the overall model performance can be clearly seen. In the microinterpretation of the SHAP model, we show the SHAP values for each feature during model evaluation separately for control participants and participants with HFpEF. In the random forest HFpEF diagnostic model, there was a relatively straightforward difference between control participants and participants with HFpEF. The SHAP model demonstrates more intuitively the discriminatory power of the random forest algorithm for participants with HFpEF. Hence, through the provision of intelligent diagnoses, our model effectively bridges the gap between health care professionals and patients, thereby addressing the prevalent issue of frequent misdiagnosis or underdiagnosis and ultimately enhancing the overall quality of care. Furthermore, users can independently access data about oral characteristics at home and transmit the data to health care professionals for expert evaluation through a smartphone app, facilitating continuous monitoring of cardiac health [39]. In addition, in underserved remote areas with limited medical resources, this portable device can be used by mobile medical teams to offer diagnostic screening services to local residents [40], thereby enhancing public health in these regions. Precision medicine and personalized health care represent the future of medical development [41], and our research can also contribute to formulating individualized treatment plans whereby the medical team can gain comprehensive insights into the patient’s condition through remotely collected digital data (Figure 5).

### Limitations

First, in this study, model construction and internal testing were conducted in only 1 center, and external testing was conducted in 1 center. Further improvement of the model for the diagnosis of HFpEF should be conducted in multiple centers. Second, this study used multiple algorithms to select the characteristic hyperspectral bands; however, we did not identify images using deep learning algorithms, which could be used for direct image analysis for HFpEF diagnosis. Third, although we used some means to reduce data redundancy and overfitting, these may still exist. Fourth, different demographic information may affect the results. In future model optimization studies, the applicability of the model in different subgroups and including easily accessible clinical information should be considered. The promotion of HSI technology in HFpEF diagnosis has prospective, broader clinical applications that need to be further explored and developed.

### Conclusion

This study demonstrates the innovative use of HSI technology to capture oral images and machine learning algorithms to construct a digital model to diagnose HFpEF. This technology was validated to have excellent performance in both internal and external testing groups. This study offers novel insights into the development of portable devices for rapid identification of HFpEF, thereby facilitating the advancement of digital diagnosis and treatment approaches for HFpEF and ultimately leading to improved patient outcomes and reduced health care costs.

## Appendix

Multimedia Appendix 1 Additional performance evaluations of the algorithms.

## Abbreviations

AUC: area under the curve
DCA: decision curve analysis
ENVI: Environment for Visualizing Images
HFpEF: heart failure with preserved ejection fraction
HSI: hyperspectral imaging
LASSO: least absolute shrinkage and selection operator
NPV: negative predictive value
PPV: positive predictive value
ROC: receiver operating characteristic
SHAP: Shapley Additive Explanations
SVM: support vector machine

## Data Availability

Individual participant data underlining the results reported in this manuscript after deidentification can be obtained from the corresponding author upon request.
