# Supplementary material for: Noninvasive Oral Hyperspectral Imaging–Driven Digital Diagnosis of Heart Failure With Preserved Ejection Fraction: Model Development and Validation Study
Source: J Med Internet Res. 2025 Jan 7;27:e67256. doi: 10.2196/67256 (PMC11751651; doi:10.2196/67256)
Supplement: Multimedia Appendix 1 [file jmir_v27i1e67256_app1.docx]

**Table S1. Performance evaluation of different algorithms in the internal testing group.**

| Machine Learning | Accuracy | F1 Score | ^a^PPV | ^b^NPV | Sensitivity | Specificity | AUC |
| --- | --- | --- | --- | --- | --- | --- | --- |
| Linear | 0.743 | 0.526 | 0.625 | 0.778 | 0.455 | 0.875 | 0.784 |
| Logistic | 0.771 | 0.600 | 0.667 | 0.808 | 0.545 | 0.875 | 0.710 |
| TheilSen | 0.686 | 0.001 | 0.001 | 0.686 | 0.001 | 0.999 | 0.750 |
| Lasso | 0.686 | 0.478 | 0.314 | 0.686 | 0.001 | 0.999 | 0.500 |
| LassoLars | 0.686 | 0.478 | 0.314 | 0.686 | 0.001 | 0.999 | 0.500 |
| MultiTaskLasso | 0.686 | 0.478 | 0.314 | 0.686 | 0.001 | 0.999 | 0.500 |
| Ridge | 0.771 | 0.625 | 0.999 | 0.375 | 0.800 | 0.625 | 0.773 |
| Lars | 0.771 | 0.645 | 0.999 | 0.250 | 0.800 | 0.625 | 0.780 |
| Orthogonal Matching | 0.800 | 0.609 | 0.999 | 0.208 | 0.833 | 0.833 | 0.758 |
| Bayesian Ridge | 0.771 | 0.636 | 0.909 | 0.333 | 0.636 | 0.833 | 0.758 |
| ^c^ARD | 0.743 | 0.693 | 0.909 | 0.458 | 0.667 | 0.917 | 0.777 |
| Tweedie | 0.771 | 0.667 | 0.999 | 0.375 | 0.615 | 0.833 | 0.795 |
| ^d^SGD | 0.743 | 0.594 | 0.600 | 0.800 | 0.600 | 0.833 | 0.735 |
| Support Vector Machine | 0.800 | 0.667 | 0.909 | 0.458 | 0.700 | 0.718 | 0.814 |
| Perceptron | 0.771 | 0.600 | 0.667 | 0.808 | 0.545 | 0.875 | 0.519 |
| ExtraTree | 0.543 | 0.385 | 0.333 | 0.700 | 0.455 | 0.583 | 0.710 |
| Kernel Ridge | 0.742 | 0.600 | 0.818 | 0.583 | 0.667 | 0.542 | 0.777 |
| Gaussian Process | 0.714 | 0.615 | 0.818 | 0.542 | 0.533 | 0.708 | 0.727 |
| ^e^PLS^e^ | 0.800 | 0.667 | 0.999 | 0.208 | 0.750 | 0.917 | 0.799 |
| Gradient Boosting | 0.771 | 0.643 | 0.818 | 0.667 | 0.667 | 0.708 | 0.777 |
| Huber | 0.743 | 0.667 | 0.999 | 0.542 | 0.667 | 0.583 | 0.803 |
| ^f^MLP | 0.743 | 0.690 | 0.909 | 0.667 | 0.600 | 0.718 | 0.765 |
| Random Forest | 0.829 | 0.750 | 0.999 | 0.542 | 0.999 | 0.792 | 0.884 |
| BernoulliNB | 0.629 | 0.519 | 0.438 | 0.789 | 0.636 | 0.625 | 0.676 |
| GaussianNB | 0.657 | 0.571 | 0.471 | 0.833 | 0.727 | 0.625 | 0.631 |
| ElasticNet | 0.686 | 0.478 | 0.314 | 0.686 | 0.001 | 0.999 | 0.500 |
| MultiTaskElasticNet | 0.686 | 0.478 | 0.314 | 0.686 | 0.001 | 0.999 | 0.500 |
| PassiveAggressive | 0.543 | 0.333 | 0.308 | 0.682 | 0.364 | 0.625 | 0.515 |

^a^PPV, Positive predictive value;

^b^NPV, Negative predictive value;

^c^ARD, Automatic relevance determination;

^d^SGD, Stochastic gradient descent;

^e^PLS, Partial least squares;

^f^MLP, Multilayer perceptron.

**Table S2. Performance evaluation of different algorithms in external testing group.**

| Machine Learning | Accuracy | F1 Score | ^b^PPV | ^c^NPV | Sensitivity | Specificity | AUC |
| --- | --- | --- | --- | --- | --- | --- | --- |
| Tweedie | 0.771 | 0.500 | 0.500 | 0.771 | 0.999 | 0.999 | 0.676 |
| Support Vector Machine | 0.800 | 0.526 | 0.600 | 0.933 | 0.875 | 0.963 | 0.792 |
| ^a^PLS | 0.771 | 0.461 | 0.429 | 0.882 | 0.875 | 0.999 | 0.671 |
| Huber | 0.771 | 0.480 | 0.375 | 0.909 | 0.875 | 0.999 | 0.634 |
| Random Forest | 0.857 | 0.625 | 0.999 | 0.844 | 0.999 | 0.999 | 0.812 |

^a^PLS, Partial least squares;

^b^PPV, Positive predictive value;

^c^NPV, Negative predictive value.**Table S3. Top 5 Best Model Feature Ranking.**

| Machine Learning | Number of Features | Feature Ranking |
| --- | --- | --- |
| Tweedie | 24 | B13-T1-S,B13-T4-S,B13-T2-S,B13-T2-M,B24-T5-M,B24-T5-M,B9-T1-S,B1-T1-S,B24-T2-M,B13-T1-M,B10-T5-M,B1-T5-S,B8-S,B4-T1-S,B4-S,B4-T8-S,B8-T5-M,B16-T5-S,B2-T4-M,B21-T5-M,B18-T8-M,B6-T8-M,B2-T5-M,B15-T5-M |
| Support Vector Machine | 7 | B13-T1-S,B13-T4-S,B13-T2-S,B13-T2-M,B24-T5-M,B24-T5-M,B9-T1-S |
| ^a^PLS | 24 | B13-T1-S,B13-T4-S,B13-T2-S,B13-T2-M,B24-T5-M,B24-T5-M,B9-T1-S,B1-T1-S,B24-T2-M,B13-T1-M,B10-T5-M,B1-T5-S,B8-S,B4-T1-S,B4-S,B4-T8-S,B8-T5-M,B16-T5-S,B2-T4-M,B21-T5-M,B18-T8-M,B6-T8-M,B2-T5-M,B15-T5-M |
| Huber | 20 | B13-T1-S,B13-T4-S,B13-T2-S,B13-T2-M,B24-T5-M,B24-T5-M,B9-T1-S,B1-T1-S,B24-T2-M,B13-T1-M,B10-T5-M,B1-T5-S,B8-S,B4-T1-S,B4-S,B4-T8-S,B8-T5-M,B16-T5-S,B2-T4-M,B21-T5-M |
| Random Forest | 25 | B13-T1-S,B13-T4-S,B13-T2-S,B13-T2-M,B24-T5-M,B24-T5-M,B9-T1-S,B1-T1-S,B24-T2-M,B13-T1-M,B10-T5-M,B1-T5-S,B8-S,B4-T1-S,B4-S,B4-T8-S,B8-T5-M,B16-T5-S,B2-T4-M,B21-T5-M,B18-T8-M,B6-T8-M,B2-T5-M,B15-T5-M,B1-T2-S |

^a^PLS, Partial least squar

**
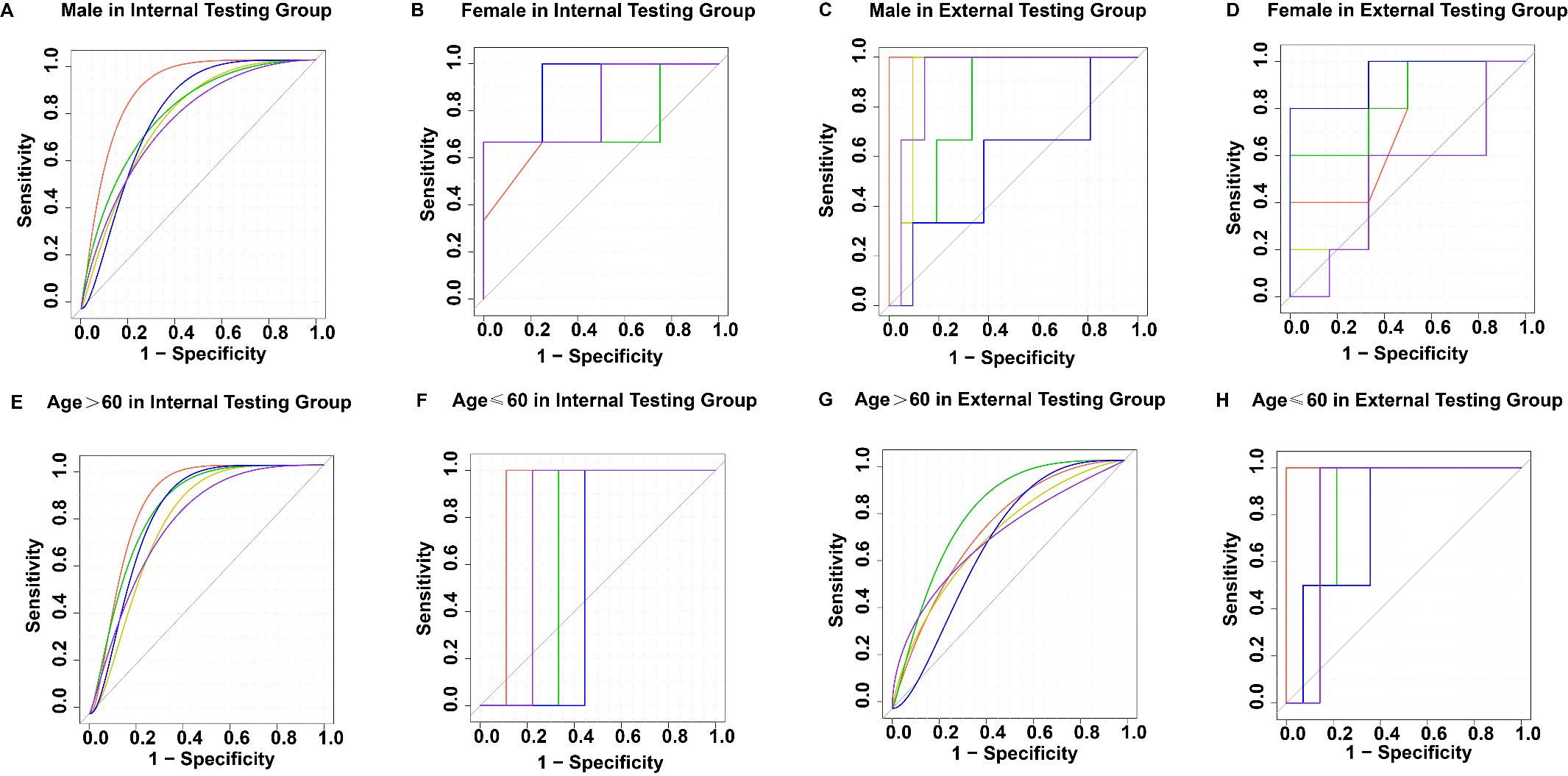
**

**Figure S1.** **ROC curves of Top 5 algorithms in subgroup analysis.** (A) ROC curve of male in internal testing group: RandomForest 0.891(95% CI 0.765-1.000), Tweedie 0.775(95% CI 0.579-0.971), SupportVector 0.794(95% CI 0.603-0.984); Huber 0.787(95% CI 0.619-0.956), PartialLeastSquares 0.756(95% CI 0.554-0.959); (B) ROC curve of female in internal testing group: RandomForest 0.875(95% CI 0.592-1.000), Tweedie 0.883(95% CI 0.456-1.000), SupportVector 0.750(95% CI 0.234-1.000); Huber 0.917(95% CI 0.686-1.000), PartialLeastSquares 0.833(95% CI 0.456-1.000); (C) ROC curve of male in external testing group: RandomForest 1.000(95% CI 1.000-1.000), Tweedie 0.921(95% CI 0.807-1.000), SupportVector 0.794(95% CI 0.594-0.993); Huber 0.571(95% CI 0.145-0.998), PartialLeastSquares 0.921(95% CI 0.803-1.000); (D) ROC curve of female in external testing group: RandomForest 0.773(95% CI 0.410-1.000), Tweedie 0.533(95% CI 0.135-0.931), SupportVector 0.833(95% CI 0.574-1.000); Huber 0.933(95% CI 0.779-1.000), PartialLeastSquares 0.500(95% CI 0.107-0.893); (E) ROC curve of age＞60 in internal testing group: RandomForest 0.863(95% CI 0.714-1.000), Tweedie 0.780(95% CI 0.583-0.977), SupportVector 0.840(95% CI 0.679-1.000); Huber 0.813 (95% CI 0.639-0.988), PartialLeastSquares 0.773(95% CI 0.580-0.967); (F) ROC curve of age≤60 in internal testing group: RandomForest 0.889, Tweedie 0.667, SupportVector 0.667; Huber 0.556, PartialLeastSquares 0.778; (G) ROC curve of age＞60 in external testing group: RandomForest 0.718(95% CI 0.448-0.989), Tweedie 0.673(95% CI 0.373-0.972), SupportVector 0.800(95% CI 0.577-1.000); Huber 0.691 (95% CI 0.393-0.989), PartialLeastSquares 0.665(95% CI 0.335-0.974); (H) ROC curve of age≤60 in external testing group: RandomForest 1.000(95% CI 1.000-1.000), Tweedie 0.896(95% CI 0.743-1.000), SupportVector 0.896 (95% CI 0.743-1.000); Huber 0.708 (95% CI 0.420-0.997), PartialLeastSquares 0.875 (95% CI 0.708-0.989).

ROC, Receiver operating characteristic
